# Psychometric validation of the 1-month recall Uterine Fibroid Symptom and Health-Related Quality of Life questionnaire (UFS-QOL)

**DOI:** 10.1186/s41687-019-0146-x

**Published:** 2019-08-23

**Authors:** Karin S. Coyne, Amanda Harrington, Brooke M. Currie, Jun Chen, Patrick Gillard, James B. Spies

**Affiliations:** 1 Evidera, 7101 Wisconsin Avenue, Suite 1400, Bethesda, MD 20814 USA; 2 Allergan plc, Irvine, CA USA; 3 MedStar Georgetown University Hospital, Washington, DC, USA

**Keywords:** Health-related quality of life, Symptoms, Uterine fibroids, Patient-reported outcomes

## Abstract

**Background:**

To evaluate the psychometric characteristics of the 1-month recall Uterine Fibroid Symptom and Health-Related Quality of Life questionnaire (UFS-QOL), including the Revised Activities subscale.

**Methods:**

VENUS I and II were phase III, randomized, double-blind, placebo-controlled trials of ulipristal acetate in women with uterine fibroids (UF) and abnormal uterine bleeding. Women completed the 1-month recall UFS-QOL at baseline and after 12 weeks’ treatment. Uterine bleeding was assessed via a daily diary (both studies); the Patient Global Impression of Improvement scale (PGI-I) was completed in VENUS II. Psychometric analyses examined internal consistency reliability and construct validity of the UFS-QOL; confirmatory factor analysis (CFA) compared model fit of the original and Revised Activities subscales. Analyses were conducted separately for VENUS I and II.

**Results:**

One hundred and fifty-seven patients in VENUS I and 429 in VENUS II were included. Changes in mean Symptom Severity and health-related quality of life (HRQoL) scale scores indicated symptom burden reductions and HRQoL improvements. Cronbach’s alpha coefficients were high at baseline and after 12 weeks’ treatment (all ≥0.76, meeting the >0.70 threshold), demonstrating strong internal consistency reliability. Correlations between UFS-QOL scores and bleeding diary responses (range: −0.35 to −0.63), and UFS-QOL scores and PGI-I responses (range: −0.48 to −0.70), ranged from moderate to strong after 12 weeks’ treatment (all *p* < 0.0001). Patients with absence of bleeding or controlled bleeding after 12 weeks’ treatment scored significantly better (*p* < 0.001) on each UFS-QOL scale than patients not achieving those end points, supporting construct validity. CFA confirmed model fit for the Revised Activities subscale.

**Conclusions:**

The 1-month recall UFS-QOL, including the Revised Activities subscale, is a valid, reliable measure to assess UF symptoms and their impact on HRQoL.

**Trial registration:**

ClinicalTrials.gov, NCT02147197. Registered May 26, 2014; retrospectively registered. ClinicalTrials.gov, NCT02147158. Registered May 26, 2014; retrospectively registered.

**Electronic supplementary material:**

The online version of this article (10.1186/s41687-019-0146-x) contains supplementary material, which is available to authorized users.

## Background

Uterine fibroids (UF) are among the most common benign neoplasms of the female pelvic region. UF incidence increases as women approach menopause and has been reported to affect up to 80% of women by the age of 50 years [1]. Up to half of women with UF experience clinical symptoms [2], including abnormal uterine bleeding (AUB) and pain [3], which can cause significant emotional and psychological distress [4]. A national survey of women in the United States aged 29–59 years with self-reported symptomatic UF revealed that 31% of respondents reported symptoms interfering with physical activities “all/most of the time”, while 22% reported symptoms interfering with daily/social activities “all/most of the time.” In addition, almost one-third of employed respondents reported missing work due to their symptoms [5].

The symptoms of UF and their negative impact on health-related quality of life (HRQoL) and activities of daily living are some of the reasons why women seek therapy for UF. Patient-reported outcome (PRO) measures are, therefore, appropriate tools to measure the impact and outcome of interventions [6]. The Uterine Fibroid Symptom and Health-Related Quality of Life questionnaire (UFS-QOL) is widely used to evaluate patient-reported UF symptoms and their impact on HRQoL, and is the only disease-specific instrument developed and validated in a population of women with UF [7]. It was developed based on qualitative input from patients with UF; the original validation demonstrated its ability to discriminate between women with and without UF and also between varying patient-reported disease severity [7]. Furthermore, the UFS-QOL has been shown to be highly responsive to change following treatment [6].

In the original version of the UFS-QOL, patients are instructed to consider their experiences with UF during the previous 3 months. The instrument has since been modified to incorporate a shorter 1-month recall period to minimize recall bias [8] and to provide a more precise assessment of treatment effect based on a monthly menstrual cycle. The availability of multiple recall versions of the UFS-QOL also provides utility for women who do not have a monthly cycle; the 3-month recall version may be more useful for women who have less frequent menstrual cycles. In addition, a Revised Activities subscale has been created to include the most relevant items pertaining to physical and social activities. This scale was developed based on recent qualitative focus groups in which participants were asked to indicate whether each item of the UFS-QOL was relevant to them. The two items that ranked lowest in terms of relevancy to patients on the Activities subscale were removed during data analysis to create the Revised Activities subscale (i.e. the items were not removed from the questionnaire itself). As a result of these changes to the UFS-QOL, further validation is warranted.

An a priori planned validation of the 1-month recall UFS-QOL, including the Revised Activities subscale, was carried out to evaluate the instrument’s psychometric properties using data from two trials of ulipristal acetate (UPA), an investigational, orally administered selective progesterone receptor modulator that reversibly blocks progesterone receptors in its target tissues (endometrium, pituitary, and UF) [9, 10]. UPA has been shown in studies to provide therapeutic effects in reducing AUB in women with UF [11–15], including two pivotal phase III trials conducted in the United States and Canada (VENUS I [UL1309; NCT02147197] and VENUS II [UL1208; NCT02147158]) [16, 17], in study populations representative of women with UF in the US general population.

## Methods

### Study designs and patients

This analysis included data from VENUS I and VENUS II, two phase III, multicenter, randomized, double-blind, placebo-controlled trials to assess the safety and efficacy of UPA for the treatment of AUB associated with UF. Both studies included pre-menopausal women aged 18–50 years who had: ultrasound evidence of at least one discrete UF; a history of cyclic (≥22 and ≤ 35 days) AUB; and menstrual blood loss ≥80 mL. Key exclusion criteria were: a history of uterine surgery that would interfere with the study end points; known coagulation disorder; and a history of, or current, uterine, cervical, ovarian, or breast cancers [16, 17]. VENUS I included 157 patients randomized to placebo, UPA 5 mg, or UPA 10 mg for 12 weeks of treatment, followed by a 12-week drug-free follow-up period. VENUS II included 432 patients randomized to placebo followed by UPA, UPA followed by placebo, or two courses of UPA. The two 12-week treatment courses were separated by a drug-free interval of two menses. The second treatment course in VENUS II was followed by a 12-week drug-free follow-up period. This report follows recommendations described in the CONSORT PRO Extension [18].

### Questionnaires and assessments

#### UFS-QOL

The 37-item, self-administered UFS-QOL measures Symptom Severity (eight items) and HRQoL (29 items) and has been previously validated [6, 7, 19, 20]. The HRQoL Total scale consists of six subscales: Concern, Activities, Energy/Mood, Control, Self-Consciousness, and Sexual Function. Response options for Symptom Severity scale items are scored from 1 (“Not at all”) to 5 (“A very great deal”); response options for items in the HRQoL subscales range from 1 (“None of the time”) to 5 (“All of the time”). The Symptom Severity scale, HRQoL subscales, and HRQoL Total scale scores are summed and transformed into a 0–100-point scale, with higher Symptom Severity scores indicating greater symptom severity and higher HRQoL scores indicating better HRQoL. The Symptom Severity scale is unidimensional and the HRQoL subscales can be treated as unidimensional scales of a multidimensional construct (HRQoL); the HRQoL Total scale is a sum of the HRQoL subscales.

Patients were instructed to consider their experiences with UF over a modified recall period of 1 month. The Revised Activities subscale, a shorter version of the original Activities subscale, was included in all validation analyses. The 1-month recall UFS-QOL was completed by patients at baseline (Visit 1; first on-treatment visit in both studies) and after 12 weeks of treatment (Visit 2; end of the 12-week treatment period in VENUS I and end of Treatment Course 1 in VENUS II), or on early withdrawal for patients who withdrew after Visit 1 and before Visit 2 (for both studies).

#### Patient Global Impression of Improvement scale

The Patient Global Impression of Improvement scale (PGI-I) is a self-administered measure used to rate patient-perceived response of a condition to therapy. The PGI-I, administered in VENUS II only, asked: “During treatment with study drug, how would you describe your menstrual/vaginal bleeding compared to before you started study drug?”. Participants responded on a 7-point Likert scale with the following options: 1, “Very much better”; 2, “Much better”; 3, “A little better”; 4, “No change”; 5, “A little worse”; 6, “Much worse”; and 7, “Very much worse”. The PGI-I was completed at Visit 2 or on early withdrawal.

#### Bleeding diary

Uterine bleeding was recorded in an electronic diary in both studies [16, 17]. A patient’s heaviest bleeding experienced over the preceding 24 h was captured by the following terms: “None”, no bleeding and no spotting; “Spotting”, evidence of minimal blood loss that does not require the use of sanitary protection (except for panty liners); “Bleeding”, evidence of blood loss that requires the use of sanitary pads or tampons; “Heavy bleeding”, more than normal bleeding relative to your experience, or the passage of clots. Absence of bleeding was defined as having no bleeding days during the last 35 consecutive days on treatment counting backward from the earlier of Day 84 or the last dose date in the treatment period (VENUS I) or in Treatment Course 1 (VENUS II). Controlled bleeding was defined as having 0 days of heavy bleeding and ≤8 days of bleeding within the analysis window (the last 56 days of treatment counting backward from the earlier of Day 84 or the last dose date in the treatment period [VENUS I] or in Treatment Course 1 [VENUS II]). No controlled bleeding was defined as having ≥1 day of heavy bleeding or ≥9 days of bleeding within the analysis window. The thresholds for bleeding, absence of bleeding, and controlled bleeding were identified a priori, based on the primary end points in the VENUS I and II clinical trials.

### Statistical analyses

Observed UFS-QOL and PGI-I scores were used in all analyses. Missing bleeding diary data were imputed consistent with VENUS I and II protocols. Scoring of the questionnaires was performed according to the developers’ guidelines. All statistical tests were two-sided and used a significance level of 0.05 unless otherwise noted. Baseline analyses were carried out on the intent-to-treat population using an observed cases approach, defined as patients who completed at least one item of the UFS-QOL at baseline. The per protocol population was defined as all randomized patients who completed the treatment period, in addition to completing at least one item of the UFS-QOL after 12 weeks of treatment (PRO approach). Descriptive analyses were performed on baseline patient demographic and clinical characteristics. Distributional characteristics of UFS-QOL scores were examined at baseline and after 12 weeks of treatment. Analyses were conducted separately for VENUS I and II in order to assess the reproducibility of the results; additionally, minor differences between the two trials were present, such as the PGI-I being administered only in VENUS II.

Psychometric analyses were conducted on the UFS-QOL, including the Revised Activities subscale, at baseline and after 12 weeks of treatment to examine internal consistency reliability and construct validity (convergent and known groups validity).

Internal consistency reliability explores associations between different items within a scale [21]. Cronbach’s coefficient alpha was calculated for each UFS-QOL scale at baseline and after 12 weeks of treatment; a value of >0.70 was considered acceptable to demonstrate internal consistency [22]. Validity refers to the extent to which an instrument measures what it purports to measure [21]. Convergent validity is the extent to which scores from the instrument are related to scores from other related instruments or concepts [21]. Spearman’s rank correlation coefficients were used to establish convergent validity between the UFS-QOL scales and bleeding diary assessments (number of bleeding days and heavy bleeding days) at baseline and after 12 weeks of treatment, and between the UFS-QOL scales and PGI-I after 12 weeks of treatment (VENUS II only).

Known groups validity is the extent to which scores from an instrument are distinguishable from groups that differ by a key indicator, often clinical in nature [20]. Known groups validity of the UFS-QOL was assessed by number of bleeding days (categorized by ≤5, >5 to 9, and >9 days), achievement of absence of bleeding, and achievement of controlled bleeding for both VENUS I and II. Known groups validity was also assessed based on PGI-I responses after 12 weeks of treatment in VENUS II. Specifically, two separate assessments were conducted based on patients’ PGI-I data: 1) by individual PGI-I score; and 2) by the collapsed PGI-I response categories of “Improved” (responses of “Very much better”, “Much better”, and “A little better”), “No change”, and “Worsened” (responses of “A little worse”, “Much worse”, and “Very much worse”).

The unidimensional factor structures of the Activities and Revised Activities subscales were examined using confirmatory factor analysis (CFA) using M*plus*. Model fit was assessed by examining three fit statistics: the Comparative Fit Index (CFI) – the model was considered to have a good fit if the CFI was ≥0.90 [23]; Root Mean Square Error of Approximation (RMSEA) – the goodness of fit of the model was considered acceptable for values <0.07 [24]; and Standardized Root Mean Square Residual (SRMR) – the goodness of fit of the model was considered acceptable for values ≤0.08 [25].

All analyses were performed in SAS version 9.4 and M*plus* version 7.4 [26, 27].

## Results

### Patients

**Fig. 1:**
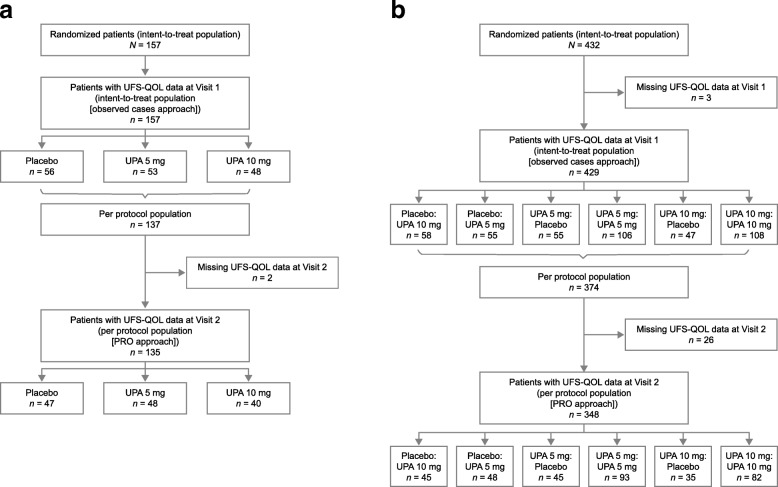
Patient populations and dispositions in (**a**) VENUS I and (**b**) VENUS II. *PRO* patient-reported outcome; *UFS-QOL* Uterine Fibroid Symptom and Health-Related Quality of Life questionnaire; *UPA* ulipristal acetate

All 157 randomized patients in VENUS I had baseline UFS-QOL data; 135 completed at least one item of the UFS-QOL after 12 weeks of treatment (or early termination) (Fig. 1). Of 432 randomized patients in VENUS II, 429 had baseline UFS-QOL data and 348 completed at least one item of the UFS-QOL after 12 weeks of treatment (or early termination) (Fig. 1). Mean (standard deviation [SD]) age was 41.1 (5.4) years and 41.0 (5.6) years in VENUS I and II, respectively. Most patients were black (68.8% in VENUS I and 66.9% in VENUS II) and mean (SD) body mass index was 31.7 (8.0) kg/m^2^ and 32.2 (7.9) kg/m^2^ in VENUS I and II, respectively (Table 1). There were no significant differences (*p* > 0.05) between women who completed 12 weeks of treatment and those who discontinued in terms of age, race, ethnicity, and body mass index.

**Table 1:** Baseline demographics (intent-to-treat population; observed cases approach)

Characteristic	VENUS I (*n* = 157)	VENUS II (*n* = 429)
Mean (SD) age, years	41.1 (5.4)	41.0 (5.6)
Race, *n* (%)		
White	46 (29.3)	130 (30.3)
Black	108 (68.8)	287 (66.9)
Asian	2 (1.3)	5 (1.2)
American Indian or Alaska Native	1 (0.6)	NR
Native Hawaiian or Other Pacific Islander	NR	2 (0.5)
Multiple	NR	5 (1.2)
Ethnicity, *n* (%)		
Hispanic and Latino	14 (8.9)	59 (13.8)
Mean (SD) body mass index, kg/m^2^	31.7 (8.0)	32.2 (7.9)

*NR* not reported, *SD* standard deviation

### UFS-QOL: scale analysis

Descriptive statistics for UFS-QOL scales are shown in Table 2. In both studies at baseline, the mean Symptom Severity scale score was relatively high (62.0 in VENUS I and 65.5 in VENUS II), decreasing to approximately half its baseline value after 12 weeks of treatment (30.5 for patients in VENUS I and 33.0 for patients in VENUS II), indicating a reduction in symptom burden. Mean scores on the HRQoL Total scale and six HRQoL subscales (and the Revised Activities subscale) at baseline ranged from 22.9 in VENUS I and 21.0 in VENUS II (both Concern) to 45.0 in VENUS I and 43.3 in VENUS II (both Control). After 12 weeks of treatment, mean scores increased, ranging from 66.1 in VENUS I (Concern) and 62.9 in VENUS II (Self-Consciousness) to 77.7 in VENUS I and 73.9 in VENUS II (both Control), indicating improved HRQoL.

**Table 2:** Distributional characteristics of UFS-QOL scale scores at baseline and after 12 weeks of treatment

	VENUS I				VENUS II			
	Baseline (*n* = 157)		12 weeks (*n* = 135^a^)		Baseline (*n* = 429^b^)		12 weeks (*n* = 347)	
UFS-QOL	Mean (SD)	Median (range)	Mean (SD)	Median (range)	Mean (SD)	Median (range)	Mean (SD)	Median (range)
Symptom Severity^c^	62.0 (19.7)	63 (0–100)	30.5 (24.5)	25 (0–100)	65.5 (21.5)	69 (3–100)	33.0 (25.8)	28 (0–100)
Concern^d^	22.9 (22.6)	15 (0–100)	66.1 (35.7)	80 (0–100)	21.0 (21.7)	15 (0–100)	66.1 (35.9)	75 (0–100)
Activities^d^	34.3 (24.6)	32 (0–100)	75.4 (29.3)	89 (0–100)	32.0 (25.7)	29 (0–100)	71.7 (32.2)	86 (0–100)
Revised Activities^d^	30.7 (24.8)	30 (0–100)	74.4 (30.5)	85 (0–100)	29.9 (25.9)	25 (0–100)	71.1 (32.9)	85 (0–100)
Energy/Mood^d^	37.3 (24.7)	32 (0–100)	74.3 (27.5)	82 (4–100)	34.6 (25.0)	32 (0–100)	69.0 (29.6)	75 (0–100)
Control^d^	45.0 (27.0)	45 (0–100)	77.7 (26.9)	85 (0–100)	43.3 (28.8)	40 (0–100)	73.9 (30.4)	85 (0–100)
Self-Consciousness^d^	35.1 (28.9)	33 (0–100)	71.4 (29.6)	83 (0–100)	31.7 (29.4)	25 (0–100)	62.9 (34.1)	67 (0–100)
Sexual Function^d^	40.3 (33.8)	38 (0–100)	72.0 (33.5)	88 (0–100)	37.7 (33.7)	25 (0–100)	66.5 (35.4)	75 (0–100)
HRQoL Total^d^	35.4 (21.4)	35 (0–100)	73.3 (26.9)	81 (4–100)	33.0 (22.3)	29 (0–97)	69.2 (29.6)	78 (0–100)

^a^*n* = 134 for Symptom Severity, Concern, Energy/Mood, Control, Sexual Function, and HRQoL Total

^b^*n* = 428 for Activities, Revised Activities, and Self-Consciousness; *n* = 427 for Concern, Energy/Mood, and Control; *n* = 426 for Sexual Function and HRQoL Total

^c^Scores range from 0 to 100, higher scores indicate greater symptom severity

^d^Scores range from 0 to 100, higher scores indicate better HRQoL. *HRQoL* health-related quality of life; *SD* standard deviation; *UFS-QOL* Uterine Fibroid Symptom and Health-Related Quality of Life questionnaire

### Internal consistency reliability

For both VENUS I and II, each of the UFS-QOL scales showed strong internal consistency at baseline and after 12 weeks of treatment, as demonstrated by the high Cronbach’s alpha values (range at baseline: 0.76 [Self-Consciousness] to 0.96 [HRQoL Total] in VENUS I and 0.79 [Self-Consciousness] to 0.96 [HRQoL Total] in VENUS II; higher alphas were reported after 12 weeks of treatment) (Additional file 1: Table S1).

### Convergent validity

In both studies, correlations at baseline between UFS-QOL scales and the number of heavy bleeding days were significant (except for the Sexual Function subscale in VENUS I), but weak, as is to be expected for correlations between objective and subjective measures (r_s_ = 0.18 [*p* < 0.05] for the Symptom Severity scale and ranging from r_s_ = − 0.15 [*p* = not significant (NS)] to −0.25 [others *p* < 0.05] for the HRQoL subscales in VENUS I; r_s_ = 0.24 [*p* < 0.001] for Symptom Severity and ranging from r_s_ = −0.16 to −0.26 [*p* < 0.001] for the HRQoL subscales in VENUS II). In VENUS I, only Symptom Severity was significantly associated with the number of bleeding days; however, the association was weak (r_s_ = 0.16; *p* < 0.05). In VENUS II, most scales were weakly, but significantly, correlated with the number of bleeding days; the Energy/Mood and Self-Consciousness subscales did not have significant correlations.

After 12 weeks of treatment in both studies, there were much stronger correlations (ranging from −0.35 to −0.63; all *p* < 0.0001) between all UFS-QOL scales and bleeding diary responses compared to baseline (Table 3). In addition, after 12 weeks of treatment in VENUS II, correlations with the PGI-I were moderate to strong [28], and were significant (all *p* < 0.0001): 0.69 for the Symptom Severity scale and ranging from −0.48 (Sexual Function) to −0.70 (Concern) for the HRQoL subscales (Table 3).

**Table 3:** Relationship (Spearman’s correlations) of UFS-QOL scale scores with bleeding diary assessments in VENUS I and VENUS II and PGI-I in VENUS II after 12 weeks of treatment; per protocol population (patient-reported outcome approach)

	VENUS I		VENUS II		
UFS-QOL	Bleeding days^a^	Heavy bleeding days^b^	Bleeding days^a^	Heavy bleeding days^b^	PGI-I
Symptom Severity	0.59	0.56	0.62	0.60	0.69
Concern	−0.61	−0.58	−0.63	−0.63	−0.70
Activities	−0.63	−0.63	−0.54	−0.58	−0.67
Revised Activities	−0.63	−0.63	−0.53	−0.57	−0.66
Energy/Mood	−0.56	−0.58	−0.44	−0.51	−0.57
Control	−0.49	−0.54	−0.46	−0.52	−0.58
Self-Consciousness	−0.43	−0.41	−0.42	−0.47	−0.53
Sexual Function	−0.44	−0.47	−0.35	−0.40	−0.48
HRQoL Total	−0.61	−0.62	−0.53	−0.58	−0.66

All *p* < 0.0001

^a^Number of days in previous 35 days – “bleeding”: days with rating of “bleeding” and “heavy bleeding”

^b^“Heavy bleeding”: days with rating of “heavy bleeding”. *HRQoL* health-related quality of life; *PGI-I* Patient Global Impression of Improvement scale; *UFS-QOL* Uterine Fibroid Symptom and Health-Related Quality of Life questionnaire

### Known groups validity

#### By bleeding diary responses

At baseline in VENUS I, patients with ≤5 days of bleeding scored significantly better (*p* < 0.05) on the Symptom Severity scale than those with >9 days of bleeding. However, pairwise comparisons showed no significant differences between groups on any of the HRQoL subscales, likely due to the small sample sizes in each group. In VENUS II at baseline, patients with ≤5 days of bleeding scored significantly better than those with >9 days of bleeding on most of the UFS-QOL scales (*p* < 0.05; except for the Energy/Mood, Self-Consciousness, and Sexual Function subscales, for which *p* = NS). Both studies showed similar trends after 12 weeks of treatment. There were significant differences on all scales between patients experiencing ≤5 days of bleeding versus those experiencing >5 to 9 days (all *p* < 0.01; both studies) and versus those experiencing >9 days (*p* < 0.05 for VENUS I, except the Self-Consciousness and Sexual Function subscales, for which *p* = NS; *p* < 0.001 for VENUS II). In VENUS II, there was also a significant difference between patients who experienced >5 to 9 days of bleeding versus >9 days of bleeding on the Revised Activities and Energy/Mood subscales (both *p* < 0.05) (data not shown).

#### By achievement of absence of bleeding and controlled bleeding

**Fig. 2:**
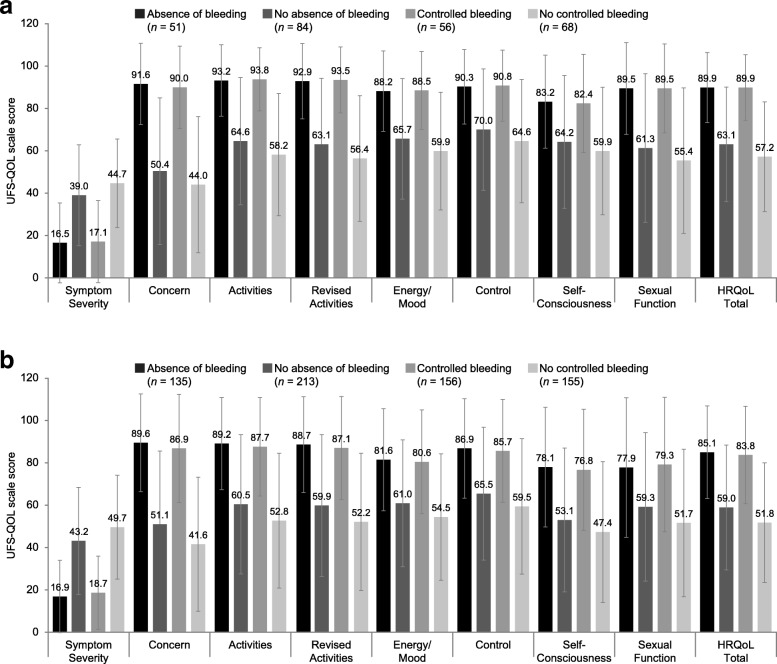
Known groups validity: UFS-QOL scale scores by achievement of absence of bleeding and controlled bleeding after 12 weeks of treatment in (**a**) VENUS I and (**b**) VENUS II: per protocol population (patient-reported outcome approach). *p* < 0.001 for comparisons of absence of bleeding versus no absence of bleeding, and controlled bleeding versus no controlled bleeding, for all scales; error bars represent standard deviation. *HRQoL* health-related quality of life; *UFS-QOL* Uterine Fibroid Symptom and Health-Related Quality of Life questionnaire

Patients who achieved absence of bleeding and controlled bleeding in both studies scored significantly better (*p* < 0.001) on each UFS-QOL scale than patients who did not achieve those outcomes (Fig. 2). For example, in VENUS II, the mean (SD) Revised Activities subscale score for women who achieved absence of bleeding compared to those who did not was 88.7 (22.7) versus 59.9 (33.5), respectively.

#### By PGI-I score

In VENUS II after 12 weeks of treatment, pairwise comparisons demonstrated that patients who responded “Very much better” on the PGI-I scored significantly better on almost all subscales when compared to each of the other PGI-I response categories. There were no significant differences on any subscale scores between those who responded “No change”, “A little worse”, “Much worse”, or “Very much worse” (data not shown; the latter three of these groups had very small sample sizes).

When PGI-I responses were collapsed into the categories of “Improved”, “No Change”, and “Worsened”, pairwise comparisons demonstrated that patients in the “Improved” group scored better on all UFS-QOL scales versus those in the “No Change” (all *p* < 0.001) and “Worsened” groups (all *p* < 0.05) (Table 4). Likely due to the small sample size and greater variance in the “Worsened” category, a monotonic pattern in scores across categories was not observed for most subscales.

**Table 4:** Known groups validity: UFS-QOL scale scores by PGI-I response categories after 12 weeks of treatment in VENUS II: per protocol population (patient-reported outcome approach)

	PGI-I						
	Improved^a^		No change^b^		Worsened^c^		Pairwise comparison
UFS-QOL	*n*	Mean (SD)	*n*	Mean (SD)	*n*	Mean (SD)	*p* value^d^
Symptom Severity	284	26.6 (21.7)	45	59.2 (22.5)	18	68.8 (23.2)	1***, 2***
Concern	285	74.7 (31.8)	44	25.2 (23.8)	18	30.6 (32.5)	1***, 2***
Activities	285	79.8 (27.2)	44	31.8 (26.5)	18	40.3 (27.7)	1***, 2***
Revised Activities	285	79.3 (27.8)	44	30.6 (27.4)	18	39.7 (29.4)	1***, 2***
Energy/Mood	285	75.2 (26.7)	44	39.4 (24.8)	18	43.2 (26.8)	1***, 2***
Control	285	79.9 (26.9)	44	47.8 (30.6)	18	42.2 (31.0)	1***, 2***
Self-Consciousness	285	68.9 (31.8)	44	31.8 (29.5)	18	42.6 (32.9)	1***, 2**
Sexual Function	285	71.3 (33.4)	44	42.6 (36.1)	18	48.6 (37.3)	1***, 2*
HRQoL Total	285	76.1 (26.1)	44	36.0 (22.7)	18	40.5 (25.7)	1***, 2***

^a^Includes responses of “Very much better”, “Much better”, and “A little better”

^b^Includes response of “No change”

^c^Includes responses of “A little worse”, “Much worse”, and “Very much worse”

^d^General linear model – pairwise comparisons between means were performed using Scheffe’s test adjusting for multiple comparisons: *p < 0.05; **p < 0.01; ***p < 0.001; 1, “improved” versus “no change”; 2, “improved” versus “worsened”. The comparison between “no change” versus “worsened” was not significant for each scale. *HRQoL* health-related quality of life, *PGI-I* Patient Global Impression of Improvement scale, *SD* standard deviation, *UFS-QOL* Uterine Fibroid Symptom and Health-Related Quality of Life questionnaire

### CFA

CFA confirmed the factor structure of the Revised Activities subscale in both studies: the CFIs exceeded 0.90 (0.97 and 0.99 in VENUS I; 0.96 and 0.98 in VENUS II for the original and Revised Activities subscales, respectively). Additionally, SRMR values indicated good model fit (0.05 and 0.03 in VENUS I; 0.03 and 0.07 in VENUS II for the original and Revised Activities subscales, respectively). Both models (in VENUS I and II) had RMSEA values slightly higher than the acceptable value of <0.07 (0.09 and 0.07 in VENUS I, both 0.10 in VENUS II); however, the RMSEA tends not to perform well in models with small degrees of freedom, as was the case here [29].

## Discussion

This psychometric validation study demonstrated that the 1-month recall UFS-QOL, including the Revised Activities subscale, is a valid and reliable PRO measure for the assessment of UF symptoms and their impact on HRQoL. The appropriate recall period for a PRO measure depends on what the measure captures, its intended use, and the attributes of the disease or study [8]. With a longer recall period, there is a risk of introducing measurement error that may reduce the chances of detecting a treatment effect [8, 30]. Given that the results for the 1-month recall version of the UFS-QOL reported here are strongly consistent with those reported in the initial 3-month recall UFS-QOL validation studies [6, 7, 19], comparison with studies using either version of the instrument is feasible.

In this analysis, the UFS-QOL was shown to detect differences between known outcomes or groups. When comparing UFS-QOL scale scores for patients who achieved absence of, or controlled, bleeding after 12 weeks of treatment versus those who did not, scores were significantly better (*p* < 0.001) for the groups who achieved those outcomes. Such discrimination of the UFS-QOL with AUB, one of the most common symptoms of UF [2], is important as it signifies that the UFS-QOL has the ability to differentiate by bleeding status.

The results of the current study corroborate the findings of an earlier validation study of the 4-week recall UFS-QOL using data from a phase IIa proof-of-concept study in 271 pre-menopausal women with heavy bleeding associated with UF [20]. Both studies provide support for the tool as a valid way to measure symptom severity and impact of UF on HRQoL. The current study is strong independently, in that it had a substantial sample size and minimal missing data. The strength of correlations between UFS-QOL scores and the number of bleeding days were weaker at baseline than after 12 weeks of treatment. These findings are comparable with those observed in the previous 4-week recall study, in which correlations between UFS-QOL scales and ratings on the Mansfield-Voda-Jorgensen Menstrual Bleeding Scale were low at baseline (<0.20), but increased after the 3-month treatment period (0.28 to 0.51; *p* < 0.0001) [20]. We would expect correlations between UFS-QOL scores and bleeding diary responses to be greater after 12 weeks of treatment compared to at baseline because the sample has changed with treatment. At the end of 12 weeks of treatment, 37.8% and 38.8% of patients were amenorrheic in VENUS I and II, respectively, with greatly improved UFS-QOL scores from baseline (Table 2); such results are reflected in the correlations between UFS-QOL scales and bleeding diary responses. In contrast, at baseline, there was much greater variability in bleeding days and UFS-QOL responses, resulting in weaker correlations.

These analyses also showed that the Revised Activities subscale performed psychometrically as well as the original Activities subscale. The Revised Activities subscale was created by removing two items ranked lowest in terms of relevancy, based on qualitative focus groups. The modified subscale showed excellent internal consistency reliability and strong model fit based on CFA results, lending support to the use of this shortened scale in future studies. While a limitation of the current study is that it was not designed to include a direct comparison of the 1-month recall UFS-QOL to the 3-month recall version of the UFS-QOL, the 1-month recall UFS-QOL demonstrated similar psychometric properties as the 3-month recall version.

## Conclusion

In conclusion, this study demonstrated that the 1-month recall UFS-QOL, including the Revised Activities subscale, is a valid and reliable PRO measure for the assessment of UF symptoms and their impact on HRQoL.

## Additional file

Additional file 1: **Table S1.** Internal consistency reliability: Cronbach’s coefficient alpha values for UFS-QOL scale scores in VENUS I and VENUS II at baseline (intent-to-treat population; observed cases approach) and after 12 weeks of treatment (per protocol population; patient-reported outcome approach) (PDF 250 kb)

## Data Availability

Allergan will share de-identified patient-level data and study-level data including protocols and clinical study reports for phase II–IV trials completed after 2008 that are registered to ClinicalTrials.gov or EudraCT, have received regulatory approval in the United States and/or the European Union in a given indication, and the primary manuscript from the trial has been published. To request access to the data, the researcher must sign a data-use agreement and any shared data are to be used for non-commercial purposes. More information can be found on http://www.allerganclinicaltrials.com/.

## Abbreviations

AUB: Abnormal uterine bleeding
CFA: Confirmatory factor analysis
CFI: Comparative Fit Index
HRQoL: Health-related quality of life
NS: Not significant
PGI-I: Patient Global Impression of Improvement scale
PRO: Patient-reported outcome
RMSEA: Root Mean Square Error of Approximation
SD: Standard deviation
SRMR: Standardized Root Mean Square Residual
UF: Uterine fibroids
UFS-QOL: Uterine Fibroid Symptom and Health-Related Quality of Life questionnaire
UPA: Ulipristal acetate
