# Supplementary material for: Psychometric validation of the 1-month recall Uterine Fibroid Symptom and Health-Related Quality of Life questionnaire (UFS-QOL)
Source: J Patient Rep Outcomes. 2019 Aug 23;3:57. doi: 10.1186/s41687-019-0146-x (PMC6708009; doi:10.1186/s41687-019-0146-x)
Supplement: Supplementary file 1 — Table S1. Internal consistency reliability: Cronbach’s coefficient alpha values for UFS-QOL scale scores in VENUS I and VENUS II at baseline (intent-to-treat population; observed cases approach) and after 12 weeks of treatment (per protocol population; patient-reported outcome approach) (PDF 250 kb) [file 41687_2019_146_MOESM1_ESM.pdf]

Table S1 Internal consistency reliability: Cronbach's coefficient alpha values for UFS-QOL scale scores in VENUS I and VENUS II at baseline (intent-to-treat population; observed cases approach) and after 12 weeks of treatment (per protocol population; patient-reported outcome approach)

	VENUS I		VENUS II	
UFS-QOL	Baseline (n = 157[a])	12 weeks (n = 135[b])	Baseline (n = 429[c])	12 weeks (n = 348[d])
Symptom Severity[e]	0.77	0.86	0.84	0.87
Concern[f]	0.84	0.96	0.84	0.95
Activities[f]	0.90	0.95	0.91	0.96
Revised Activities[f]	0.90	0.95	0.91	0.95
Energy/Mood[f]	0.91	0.95	0.91	0.94
Control[f]	0.85	0.92	0.88	0.92
Self-Consciousness[f]	0.76	0.81	0.79	0.85
Sexual Function[f]	0.87	0.91	0.83	0.90
HRQoL Total[f]	0.96	0.98	0.96	0.98

[a]n = 157 for Concern, Energy/Mood, Control, and Sexual Function; n = 156 for Self-Consciousness; n = 155 for Activities and Revised Activities; n = 154 for HRQoL Total; n = 153 for Symptom Severity

[b]n = 134 for all scales and subscales

[c]*n* = 428 for Symptom Severity; *n* = 426 for Self-Consciousness; *n* = 425 for Concern; *n* = 424 for Energy/Mood, Control, and Sexual Function; *n* = 421 for Activities and Revised Activities; *n* = 412 for HRQoL Total

[d]*n* = 346 for Concern, Self-Consciousness, and Sexual Function; *n* = 345 for Symptom Severity, Activities, Revised Activities, Energy/Mood, and Control; *n* = 339 for HRQoL Total

[e]Scores range from 0−100, higher scores indicate greater symptom severity

[f]Scores range from 0−100, higher scores indicate better HRQoL

HRQoL health-related quality of life; *UFS-QOL* Uterine Fibroid Symptom and Health-Related Quality of Life questionnaire
